# Adapting an Expanded Access program to enable investigational treatments for COVID-19

**DOI:** 10.1017/cts.2022.403

**Published:** 2022-05-16

**Authors:** Elias Samuels, Ellen Champagne, Misty Gravelin, Jamie Racklyeft, Kevin Weatherwax

**Affiliations:** 1 Michigan Institute for Clinical & Health Research, University of Michigan, Ann Arbor, MI, USA; 2 College of Pharmacy, University of Michigan, Ann Arbor, MI, USA

**Keywords:** Retrospective case studies, Clinical Translational Science Award, CTSA, Expanded Access, COVID-19

## Abstract

Retrospective case studies of initiatives supported by the National Institutes of Health’s Clinical and Translational Science Award (CTSA) hubs can be used to identify facilitators and barriers of translational science. This case study investigates how a CTSA Expanded Access program adapted to changing FDA guidance issued in 2020 to support clinicians’ treatment of COVID-19 patients in Michigan. We studied how this program changed throughout the pandemic to support physicians’ requests for remdesivir, convalescent plasma, and other uses of unapproved drugs and novel medical devices. A protocol for retrospective translational science case studies of health interventions developed by CTSA evaluators was used for this case study. Data collection methods included seven interviews and a review of institutional data, peer-reviewed publications, news stories, and other public records. The barriers identified include evolving guidance, misalignment of organizational operations, and the complexity of the research infrastructure. The facilitators of translation include collaboration between research and care teams, increasing engagement with a broad network of supporters, and ongoing professional development for research staff. The findings of this case study can be used to inform future investigations of the principles underlying the translational process.

## Introduction

The Michigan Institute for Clinical & Health Research (MICHR) helped University of Michigan (U-M) physicians provide cutting-edge treatment to severely ill COVID-19 patients in 2020 by effectively adapting its Expanded Access (EA) program to rapidly and repeatedly changing federal guidance. Health care and research teams used collaborative problem solving to support U-M physicians’ requests to administer remdesivir, convalescent plasma, and additional uses of unapproved drugs and novel medical devices to help treat dozens of critically ill COVID-19 patients in the first year of the pandemic. These teams overcame barriers to the access of investigational drugs and devices by coordinating access to key institutional resources and facilitating physicians’ treatment of patients hospitalized in the first two waves of the pandemic in Michigan. Lessons learned from this and other studies of EA programs are being disseminated through a collaborative U01 Award led by MICHR to promote EA initiatives nationwide.

This retrospective case study regards one EA program administered by MICHR, a Clinical and Translational Research Award (CTSA) institute funded by the National Institutes of Health (NIH) to advance clinical and translational science. A protocol for translational science case studies was followed to enable cross-case analysis of the barriers and facilitators of translational processes [1,2]. The results demonstrate how health care and research teams at U-M adapted the operations of the EA program in ways that were sustainable, collaborative, equitable, flexible, managed for effective and efficient performance, and proportionate to relevant risks [3–6].

Configurational comparative methods were used to identify barriers and facilitators of the translational processes that governed the accessibility of investigational treatments for patients during the COVID-19 pandemic [7]. Health care and research teams supported U-M physicians’ EA requests for COVID-19 treatments by coordinating access to critical care resources required to mitigate the local impact of the pandemic. This coordination of support contributed to the development of a learning health system by integrating best practice knowledge directly into a decision-making process governing physicians’ engagement of this EA program [8]. More broadly, the findings of this case study add to a growing body of research demonstrating how CTSA institutes have helped to mitigate the effects of COVID-19 [9–13].

## Regulatory Pathways: Expanded Access and Emergency Use Authorization

The EA pathway allows for the use of investigational drugs, devices, and biologics for the clinical treatment of patients with serious or life-threatening conditions and no satisfactory therapeutic options. Per FDA regulation 21 CRF 312.305(1)(3), the use of these products through EA may not interfere with commercial development, including preventing or discouraging the enrollment of eligible patients into clinical trials.

This pathway can take two forms: single patient or group access. Single patient EA requires a two-page submission to the FDA (Form 3926) and approval of at least the Institutional Review Board (IRB) Chair, depending on the details of the situation. This is often used in emergency cases, as verbal or email authorization from the FDA and IRB Chair can allow treatment until the paperwork is completed. Group access, typically deemed EA Programs, are more structured treatment protocols that include eligibility criteria and an Investigational New Drug Application or Investigational Device Exemption submission to the FDA, as well as IRB approval at each site.

Although similar in name and function to an EAP, an Emergency Use Authorization (EUA) is a separate pathway to access unapproved drugs, devices, and biologics for clinical use. EUAs are a form of temporary marketing authorization, akin to the commercial approval of the product, that allows the limited sale and use of the drug, device, or biologic during a period of public health emergency. These authorizations do not need IRB approval at a site but do require that the patient be provided with detailed information (in the form of a Fact Sheet) and be informed that the product has not been fully approved. Due to the conditions of the public health emergency, federal or state authorities may place additional requirements as a measure of risk mitigation, which could include supply constraints, restrictions on use, or data collection on safety and effectiveness.

The operation of EA programs requires the involvement of the research infrastructure of academic medical centers like U-M’s [14]. Navigating the changing pathways for access requires specialized knowledge of regulatory process that is not readily available in the clinical operations of academic medical centers but is often found within their research enterprise. The research enterprise of Michigan Medicine, the academic medical center of the University of Michigan, was critical to the establishment of its EA program.

## The Establishment of MICHR’s EA Program

The development of MICHR’s EA program preceded the onset of the COVID-19 pandemic by several years. Informal EA support was offered as early as 2009. The creation of an Expanded Access Oversight Committee at Michigan Medicine in 2015 marked a key milestone in the development of this program. At this time, key stakeholders recognized that the availability of a regulatory support office to guide physicians through the EA process was a potentially beneficial structure for ensuring equitable access to EA support resources [15]. The following year, a dedicated IRB application was developed and formalized infrastructure for EA support was established within Michigan Medicine.

In 2017, a formal process for executing agreements between manufactures and the EA program was put into place and the medical school’s clinical trials support infrastructure was engaged in the operation of the program. Further funding was obtained in 2018 through a supplementary award from NCATS for Transforming Expanded Access to Maximize Support and Study (TEAMSS), a multi-site effort aimed at developing a federated, national consortium for EA interventions to advance clinical care and translational research by improving patient access to experimental therapies. At this point, key stakeholders in the EA program advocated for the use of the FDA’s EA pathway (as opposed to the Right to Try Act) to provide critically ill patients with investigational medical products through a system that makes patient safety and care paramount [16].

In the years before the COVID-19 pandemic began, the process used by Michigan Medicine physicians to request EA support was standardized and aligned with IRB operations. The CTSA hubs involved in the TEAMSS award began to disseminate resources and best practices for EA to other academic medical centers in the CTSA Consortium and beyond. Physicians’ demand for EA support increased exponentially during this period. By the end of 2019, MICHR’s EA program was well established with a proven track record processing 436 requests for support over the past decade, including 337 requests for investigational drugs, 72 requests for medical devices, and 27 for biologics (Table 1).

**Table 1.** Expanded Access requests supported (2009–2019)

Year	Device	Drug	Biologic	Total
2009		1		1
2010		5		5
2011	1	2		3
2012	1	9		10
2013	1	15		16
2014	4	18		22
2015	7	17		24
2016	7	24	4	35
2017	13	44	8	65
2018	20	66	6	92
2019	18	136	9	163

This case study demonstrates how MICHR’s EA program contributed to mitigating the impact of the COVID-19 pandemic in the fall of 2020. A multi-site SMARTIRB was obtained (IRB-2017-2018 CR00004870) by six CTSA hubs using a common case study protocol [1]. Staff members provided background and basic information about the program, in addition to a list of potential interview subjects. Data were collected from semi-structured interviews, MICHR records and protocols, institutional data sources, peer-reviewed publications, news stories, and other public records that were also collected for the case study file. An infographic timeline was developed providing a depiction of the impact of MICHR’s EA program on human health, showing dozens of COVID-19 patients received EA services as well as key scientific works that were published.

Thirteen people, both faculty and staff, were invited to participate in interviews to share diverse perspectives and knowledge about different aspects of the EA process of which seven were accepted. These interviews involved four faculty and three staff at U-M and were all held during the first quarter of 2021 via Zoom. Interviews were transcribed and coded by three staff members separately with discrepancies reconciled in subsequent meetings. Three trained raters used the Rigorous and Accelerated Data Reduction (RADaR) technique to code and analyze the interview transcripts and case study records [17].

## Case Study Findings

The case study presented here begins with the arrival of the COVID-19 pandemic in Michigan. Even before the first sick patient arrived at the institution, there was recognition among the stakeholders of MICHR’s EA program that it would need to be adapted and utilized to mitigate the health impact of the pandemic. In February of 2020, MICHR’s EA program was charged to prepare to support physicians’ requests for the treatment of COVID-19 patients. Shortly afterwards, on March 10, the first case of COVID-19 was discovered in Michigan. One day later, the World Health Organization declared the disease a global pandemic.

During 2020, Michigan endured two dramatic surges of COVID-19 throughout communities across the state. In Washtenaw County, where U-M Ann Arbor is located, over 300 EA services were provided to support physicians at the U-M hospitals, including dozens of services associated with COVID-19 cases. The EA program supported physicians caring for COVID-19 patients throughout this period even as CDC and FDA guidance for their treatment changed dramatically.

In March 2020, remdesivir, convalescent plasma, and other therapies for COVID-19 patients were only available through single-patient EA, and the EA program continued providing the standard support offered before the pandemic. In early April, when the Mayo Clinic EA program for convalescent plasma became available, the EA program pivoted to provide support for relevant data reporting and complying with regulatory requirements. Remdesivir became accessible to Michigan Medicine patients through clinical trials being conducted at the university. The FDA issued an EUA for remdesivir in May, and the EA program redirected once again to provide support for physicians who requested help with mandated reporting. This would continue until approval of remdesivir in October.

During 2020, the EA program provided over 300 services, including 158 consultations (75 of which were for COVID-19 cases) and 151 lifecycle maintenance submissions (nine of which were for COVID-19 cases). The lifecycle maintenance provided included communication with drug or device manufacturers, preparation, and submission of the applications to the FDA, preparation and submissions to the IRB, coordination of the appropriate services in Research Pharmacy, and the central administration of these applications on behalf of the physician. Table 2 shows the monthly tally of EA services provided throughout the year, including for COVID-19 cases. The majority (70%) of these services were requested by physicians at the university and 20% were requested by other university faculty. Five of the 75 clinicians supported by this program were also junior faculty members at the university. The success of the EA program in supporting the treatment of COVID-19 patients was also promoted in news stories published by MICHR during this period [18]. Most importantly, MICHR’s EA Program helped physicians provide cutting-edge treatments to over 80 critically ill patients throughout the first wave of the pandemic. The ultimate impact of MICHR’s adaption of its EA program must be measured in terms of the health of individual patients like these.

**Table 2.** Expanded Access services supported throughout 2020

Services	Jan	Feb	Mar	Apr	May	Jun	Jul	Aug	Sep	Oct	Nov	Dec	Total
Expanded Access consultations	14	7	12	12	8	11	27	14	21	22	3	7	158
COVID-19 cases			2	6	3	7	16	9	15	17			75
Expanded Access lifecycle/other submission	23	15	18	11	17	12	20	15	5	5	5	5	151
COVID-19 cases				1	2	3	3						9
Total services	37	22	30	23	25	23	47	29	26	27	8	12	309

Analyses of the case study records, interview transcripts and codes resulted in the identification of three barriers and facilitators to the translational process. Ultimately, 15 codes were used in the transcripts. The three most used codes (*N* = 77) derived from the transcripts include references to the EA program as critical infrastructure (18%), the EA programs’ provision of key expertise and knowledge (15%), and MICHR filling operational gaps within U-M. Table 3 details the type of health intervention being studied, a list of milestones, and key themes and outcomes of the case study [19].

**Table 3.** Classifications, milestones, themes and outcomes of the case study

Variable	Evidence
Type of intervention*	(3) Other forms of intervention
	(c) Implementation research
Disease	Severe acute respiratory syndrome coronavirus 2 (COVID-19)
Populations affected	COVID-19 patients admitted to the University of Michigan hospitals in 2020
Key milestones:	Expanded Access (EA) oversight committee chartered; Dedicated CTSA; Institutional Review Board (IRB) and contracting infrastructure established; Clinical research unit engaged; TEAMSS U01 awarded; Standardized & aligned EA request & IRB processes; Dissemination of EA program beyond the university; EA support for COVID-19 patients requested
Key themes-barriers	Evolving FDA guidance; Misalignment of organizational operations; Complexity of the relevant research infrastructure
Key themes-facilitators	Collaborative problem solving around shared goals; Increasing engagement with a broad network of supporters; Ongoing professional development for research staff
Outcomes achieved	EA program adapted to new federal guidance; scientific & implementation studies published; COVID-19 patients treated with investigational therapies
Translational stages covered by the case**	T1, T2, T3, T4

* Smith PG, Morrow RH, Ross DA, editors. Field Trials of Health Interventions: A Toolbox. 3rd edition. Oxford (UK): OUP Oxford; 2015 Jun 1. Chapter 2, Types of intervention and their development. Available from: https://www.ncbi.nlm.nih.gov/books/NBK305514/ [19].

** NCATS Translational Stages: T0=Basic Research, T1=Preclinical Research, T2=Clinical Research, T3=Clinical Implementation and T4=Public Health (full definitions are found at https://ncats.nih.gov/translation/spectrum).

## Barriers to Translation

### Evolving Guidance for the Treatment of COVID-19 Patients

The guidance on pathways for obtaining COVID-19 treatments changed multiple times over the course of 2020 due to evolving circumstances. This proved to be a challenge to the EA program’s ongoing operations during the pandemic. In the words of a clinician involved with the EA program, this changing environment required the program personnel “to be able to figure it out as you go and do a lot of, basically, judgment calls about where the wind is blowing.” From the point of this physician, the evolving changes to guidance made by the FDA over the course of the year challenged the ability of teams supporting the EA program to adapt.

“Specifically, through the summer, I think information sharing and information overload was an issue. The FDA was constantly issuing new guidance, new guidance documents, they would approve remdesivir, and not approve it, but they said it had Emergency Use Authorization, then it didn't. I just think keeping up with the bombardment of information about the pandemic, about drugs and devices to treat the pandemic, about the whole Emergency Use Authorization process and what that entailed and what that meant, that was all new to us, we didn't have experience with that in the past.”

### Misalignment between the Operations of Internal and External Organizations

One barrier encountered by the EA program faculty and staff regarded the misalignment of their operations with the external organizations with which they had to partner. While the EA program was successful in adapting its practices to respond to changing guidance, the administrators reported that many external organizations they partnered with were not able to change their operations in response to the conditions of the pandemic as quickly for a variety of reasons. This challenge served to exacerbate the view, in the words of one clinician that, “[many] don't think that’s anything within anyone’s local control.” Or, at minimum, that “certain parts of it are out of the EA program or MICHR or University of Michigan’s control.”

Considerable ongoing effort was therefore required in collaborations with external organizations to ensure their requirements were aligned with the current restrictions faced by clinicians and the EA program. As one physician supported by the EA program described,

“In order to obtain Expanded Access, you have to work carefully with the company and have a contact with companies that will be providing the drug or device, and that was a challenge in the past for whomever happened to be. … It’s overcome more successfully because now that we have staff members who are dedicated to helping all Expanded Access here at University of Michigan, they have established contacts, they know who to contact, what companies, what they might require, they might require a specific request form that you complete initially, or they might have an online portal. Before it was Googling to haphazardly find out a lot of that information, and now [EA Program administrators] have acquired their own logs and databases and systems in place to know which companies, especially the more major companies that we work with, the manufacturers that we work with, what the process is, and how best to get that process started.”

### Complexity of the Research Infrastructure Enabling Access to Investigational Drugs and Devices

Another barrier encountered in adapting the EA program to changing FDA guidance for the treatment of COVID-19 patients was the complexity of the relevant research infrastructure within and outside of the university. As one clinician noted, “one big hurdle that [this EA program] helped to overcome,” for the “busy clinician who might be on service with 20 patients and one patient who needs the drug,” was to provide regulatory support and expertise that enabled them to access treatments through EA pathways despite the complexity of the health care and research systems that had to be involved.

The effort required by physicians to navigate the complex EA infrastructure was mitigated by the integration of the EA program into the existing research infrastructure of the university. This infrastructure includes the FDA, drug or device manufacturers, U-M’s Institutional Review Boards, Michigan Medicine’s Research Pharmacy, Office of Research, MICHR, and the university’s clinical enterprise involved in the care of the patients for which treatments are being accessed. In early 2020, this infrastructure was quickly leveraged to meet dramatically increasing demand for EA support from clinicians treating COVID-19 patients at the university hospital. As one physician noted,

“The [EA] program knew a lot about how to work through that flow chart that I wouldn't be able to do without the program. … There are communications with the FDA, there’s sign-off that you have to do. And then interactions with the company, in this case, Gilead, there are interactions with the patient, there are interactions with the pharmacy in terms of receipt and dispensing, and then there’s documentation. And so, it’s a very complicated process, and a lot of moving parts and different people who are involved, and so the coordination appears critical because there’s no way I would be able to do it as a treating physician. There’s absolutely no way I could have gotten anything done unless someone basically held my hand and walked through the process and said, ‘Sign here, do this, call this person, write this thing in my chart,’ that level of just figuring out the process.”

## Facilitators of Translation

### Collaborative Problem Solving of Research and Care Teams Around Shared Goals

The collaborative problem solving of the care and research teams around shared goals enabled the provision of effective EA support while new COVID-19 treatments were being introduced. One program administrator believed that it was “extremely critical” that the teams enabling EA support, “from the IRBs and from the research administrators and from everybody,” shared a commitment to “work together to make it happen.”

Similarly, a clinician who requested EA support described working with teams that were, “able to problem solve together rather than working to the same end on opposite sides of the wall,” because those involved were, “collaborative and congenial here across the ranks.” One described how the shared work of the university’s research and care teams to treat severely ill COVID-19 patients helped them see the importance of collaboration between research and care teams in even sharper relief.

“I think it has really renewed my perspective that having robust resources to assist with both regulatory hurdles, but also information barriers, is critically important. So, for clinicians who are very busy and inundated with patients and pressing clinical matters, being up to date on what EA looks like, distinguishing that from other means of accessing experimental interventions and knowing who to call, when to call them, and what to do in order to get patients what they need it is critically important, and having the necessary resources to do so is a huge advantage. And part of my involvement in this project has been realizing that this is not purely a matter of ethics, it’s also a matter of logistics and manpower and resources, which also, of course, go hand in hand.”

### Deepening Engagement with a Broad Network of Stakeholders

Ongoing stakeholder engagement within and outside of the university facilitated the work required to provide consistent EA support throughout the first year of the pandemic. The relationships that faculty and administrators of the EA program cultivated with key stakeholders enabled quicker communication and effective coordination between teams working in the clinical care and research enterprises of the university. This stakeholder network included numerous physicians, faculty, and staff at U-M’s IRB, Research Pharmacy, MICHR, and clinical departments throughout the health system. Externally this network included physicians, researchers, and administrators at other clinical research organizations and academic medical research centers, as well as current and former employees of the FDA.

Clinicians and administrators supported by the EA program described how the close and collegial relationships cultivated by this network were essential to the timely adaptation of the EA program for the treatment of COVID-19 patients. One EA program administrator similarly noted that,

“We [had the] support from the IRBs and from the research administrators and from everybody saying, ‘Okay, yes, you can do this. We will work together to make it happen. Contact [the EA Program Administrator]. We’ll get you in contact with the IRB. We will do everything that we can do.’ And really making it a priority. And I know it’s a priority throughout the whole university, and it needed to be… If I said, “Oh, I’m going to call [the IRB] and we’ll see how we’re going make this happen.” I knew that [the IRB Director] would make that the highest priority because this was a COVID patient, and we were working to get this through. I think it’s extremely important if we were just one little cog trying to convince everybody else, it wouldn't have been as successful as it was if the whole research infrastructure didn't work to support this Expanded Access during the pandemic.”

### Ongoing Professional Development within MICHR’s EA Program

Support for the ongoing professional development of research staff proved to be a crucial facilitator enabling the EA program to adapt to changing guidance for COVID-19 treatments. Key research staff involved in the EA program accrued experience with FDA regulations years in advance and developed foundational professional skills. One EA program administrator described how cross-training two staff members in the skills involved in providing standard expanded access support was required to grow and adapt the program during the pandemic. This cross-training was also described as being beneficial to the administrators’ ongoing professional development and potential for advancement.

The importance of teams of individuals developing skills and knowledge before and during the pandemic was critical to the adaptation of the program to the changing guidance. Developing this expertise across staff in the EA program enabled it to change its operations in response to the dramatic increases in demand for physicians’ requests for support. As one administrator noted,

“For quite a while during the summer, because [administrator A] was so busy working just on remdesivir and convalescent plasma reporting that was required for each single patient, … all of the other requests were going to [administrator B] who was even working some over time just to try to take the rest of the Expanded Access work that [administrator A] would have normally been doing, because [they] was so busy doing all of this COVID-19 remdesivir and convalescent plasma required reporting.”

## Current Status of Impact, Dissemination, and Implementation

**Fig. 1.**
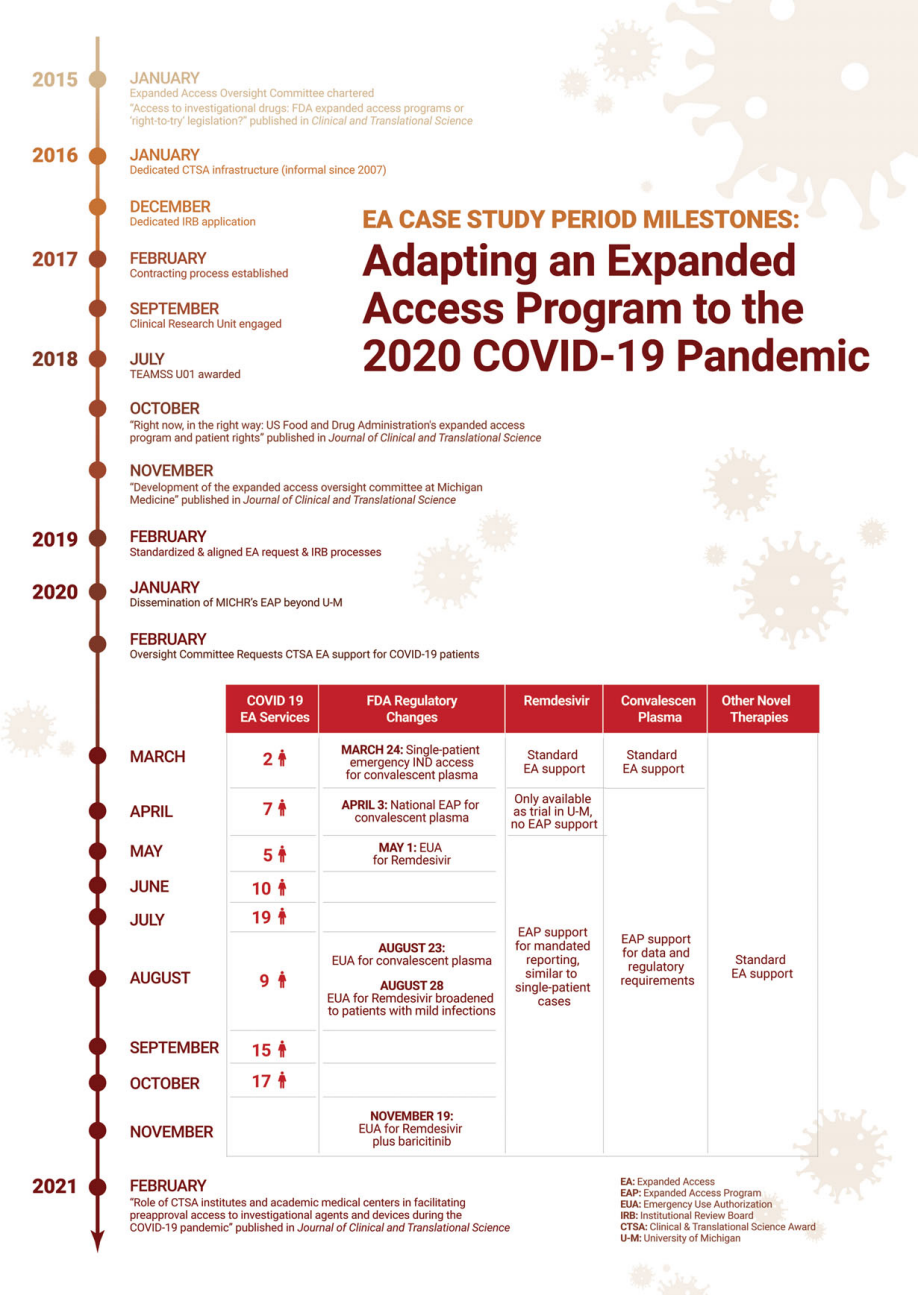
Timeline of adapting MICHR’s Expanded Access program to COVID-19 guidance.

The EA program at U-M continues to support clinicians who are treating severely ill COVID-19 patients. In 2020, MICHR’s EA program supported 84 COVID-19 cases, providing physicians with critically ill patients access to cutting-edge medical treatments for the new virus. And the lessons learned by EA programs operating during the pandemic at multiple CTSA hubs, including MICHR, have been published. Figure 1 shows how these impacts accrued over the course of the year in parallel with changes in FDA guidance for the treatment of COVID-19. Institutional and federal funding will be used to make further investments in the clinical and research infrastructure required for adaptable and effective EA programs at U-M and at other academic medical centers in the CTSA Consortium. This ongoing support is essential to building the administrative and scientific capacity required to adapt this and other EA programs to evolving FDA guidance for COVID-19 treatments.

The health care and research teams who continue to support U-M physicians’ EA requests are applying the lessons learned during the pandemic to better govern access to critical care resources and to disseminate best practices to similar programs nationwide. Their work demonstrates that regulatory knowledge and expertise must be deeply embedded within institutions in order for them to adapt to a sudden public health crisis, and efficient systems for accessing experimental therapeutics and flexibility in the governance of institutional resources are necessary to enhance patient access to investigational drugs and devices [14]. By applying and disseminating their lessons learned, these collaborating teams are helping to foster the development of learning health systems within and outside of the university [8].

Adapting U-M’s EA program to changing pathways for the treatment of COVID-19 also led indirectly to new translational research aimed at further mitigating the effects of the pandemic, as described by one individual. The way that this process of adaptation created the opportunity to advance clinical research across the translational spectrum was detailed by an administrator involved with the program.

“There’s a [this] device, it’s a device that’s attached to dialysis and ECMO, and that device has been developed here at U-M for other indications. There are ongoing research studies here for other indications, and our investigators here thought that it would work [for] some of these severely ill COVID-19 patients who are on ECMO. And so, they reached out to us, and we were able to obtain Expanded Access use for that device for several of our patients here, and it did so well that the company who makes this device has set up an actual clinical trial to study this device in COVID-19 patients. … So, I know that’s a particular success that was only available because of the Expanded Access use here. To my knowledge, there are several publications in which they wrote about those cases.”

## Conclusion

The CTSA Consortium has long aimed to advance translational science in ways that yield more medical treatments, more quickly. Shortly after the start of the COVID-19 pandemic, the National Center for Advancing Translational Sciences amended the CTSA funding opportunity announcements to require CTSAs to develop strategic plans that leverage local adaptive capacities to address emergent research needs and that impact the clinical and translational science enterprise. Our understanding of the field of translational science can be advanced by case studies like this one, which identify barriers and facilitators to translational processes, such as ones that affect the equitable and timely access to investigational drugs and devices during public health emergencies.

Case studies of translational health interventions like this one can advance the fields of translational and team science by focusing on the mechanisms that cultivate learning health systems [8]. This case demonstrates how the health care and research teams involved in the adaptation of MICHR’s EA program utilized competencies that characterize successfully translational teams, specifically including collaborative problem-solving skills and team learning behaviors [20]. The results of this case also suggest that these teams’ understanding of the organizational, managerial, and scientific complexities involved in their work was essential to their collaborative adaptation of this translational program [21]. And, the individuals on these teams shared many of the characteristics of translational scientists, particularly including possessing deep disciplinary knowledge and expertise, and working collaboratively outside of organizational silos to advance medical interventions [22].

The contributions that MICHR’s EA program made to mitigating the health effects of the COVID-19 pandemic were enabled by the collaborative approach taken by the health care and research teams charged with adapting their work to a rapidly evolving environment. The way in which they collaborated enabled these separate teams to operate as a network without which vital translational treatments would have been unavailable. Future research should extend this case by studying the long-term effects of these types of collaborative efforts to mitigate the impact of the pandemic on the structure and operations of academic medical centers.

By adapting its EA program to support physicians’ requests for COVID-19 treatment, MICHR ensured regulatory support for dozens of physicians providing cutting-edge treatment to COVID-19 patients in 2020. The key barriers and facilitators of the translational process described here affected the EA program’s support of physicians’ access to investigational treatments for a new disease. Adapting this program to rapidly evolving FDA guidance for the safe and effective treatment of COVID-19 patients enabled access to cutting-edge therapies used to treat critically ill patients hospitalized in the earliest waves of the pandemic in Michigan.
